# Clinical stage drugs targeting inhibitor of apoptosis proteins purge episomal Hepatitis B viral genome in preclinical models

**DOI:** 10.1038/s41419-021-03924-0

**Published:** 2021-06-23

**Authors:** Michelle P. Clark, Thao Huynh, Shringar Rao, Liana Mackiewicz, Hugh Mason, Shahla Romal, Michael D. Stutz, Sang H. Ahn, Linda Earnest, Vitina Sozzi, Margaret Littlejohn, Bang M. Tran, Norbert Wiedemann, Elizabeth Vincan, Joseph Torresi, Hans J. Netter, Tokameh Mahmoudi, Peter Revill, Marc Pellegrini, Gregor Ebert

**Affiliations:** 1 The Walter and Eliza Hall Institute of Medical Research, Parkville, VIC Australia; 2 Department of Medical Biology, The University of Melbourne, Parkville, VIC Australia; 3 Victorian Infectious Diseases Reference Laboratory, The Royal Melbourne Hospital at The Peter Doherty Institute for Infection and Immunity, Melbourne, VIC Australia; 4 Erasmus University Medical Center, Rotterdam, The Netherlands; 5 Department of Internal Medicine, Yonsei University, Seoul, South Korea; 6 Department of Microbiology and Immunology, The University of Melbourne at The Peter Doherty Institute for Infection and Immunity, Melbourne, VIC Australia; 7 Department of Infectious Diseases, The University of Melbourne at The Peter Doherty Institute for Infection and Immunity, Melbourne, VIC Australia; 8 Debiopharm International S.A., Lausanne, Switzerland; 9 Curtin Medical School, Curtin University, Perth, WA Australia; 10 Present Address: Vaccine and Gene Therapy Institute, Oregon Health and Science University, Beaverton, OR USA; 11 Present Address: Institute of Virology, Technical University of Munich/Helmholtz Zentrum München, Munich, Germany

**Keywords:** Target validation, Hepatitis B, Preclinical research, Translational research

## Abstract

A major unmet clinical need is a therapeutic capable of removing hepatitis B virus (HBV) genome from the liver of infected individuals to reduce their risk of developing liver cancer. A strategy to deliver such a therapy could utilize the ability to target and promote apoptosis of infected hepatocytes. Presently there is no clinically relevant strategy that has been shown to effectively remove persistent episomal covalently closed circular HBV DNA (cccDNA) from the nucleus of hepatocytes. We used linearized single genome length HBV DNA of various genotypes to establish a cccDNA-like reservoir in immunocompetent mice and showed that clinical-stage orally administered drugs that antagonize the function of cellular inhibitor of apoptosis proteins can eliminate HBV replication and episomal HBV genome in the liver. Primary human liver organoid models were used to confirm the clinical relevance of these results. This study underscores a clinically tenable strategy for the potential elimination of chronic HBV reservoirs in patients.

## Introduction

Approximately 260 million people are chronically infected with HBV and are at increased risk of developing liver cancer compared to the general population [1]. Current antiviral nucleoside and nucleotide analogs, such as entecavir (ETV), efficiently block HBV reverse transcription to suppress HBV replication, but lifelong adherence is required to maintain aviremia. A hallmark of HBV infection is the generation of a stable persistent cccDNA viral episome, referred to as a mini-chromosome, in the nucleus of infected hepatocytes. HBV cccDNA is the template for the transcription of HBV RNAs and is an integral component of the viral replicative lifecycle. Presently no drugs have been shown to effectively eliminate HBV cccDNA which is considered to be a major obstacle to curing chronic HBV infection and reducing the risk of hepatocellular carcinoma [2]. The ultimate goal is to develop strategies that efficiently eliminate HBV cccDNA in an effort to potentially cure chronic HBV infection [3].

Robust preclinical models of chronic HBV that allow the study of cccDNA dynamics would greatly facilitate endeavors to explore novel therapeutic options [4]. Immunocompromised mice with transplanted human livers permit viral entry [5] and are useful tools to study nuclear cccDNA [6]. The lack of a functional immune system in these animals, however, limits the ability to explore therapeutic modalities that rely on immune signaling networks. Various adenovirus and adeno-associated virus vectors have been used to deliver HBV DNA to mouse hepatocytes on an immunocompetent background [6–8]. A shortcoming of these vectors is that they transduce many tissues beyond the liver and induce innate and adaptive immune responses independently of HBV, which confounds the interpretation of therapeutic studies [9]. AAV-HBV-transduced mice allow for limited qualitative or quantitative assessments of episomal HBV cccDNA or their equivalents [10]. Hydrodynamic injection (HDI) of plasmid DNA represents an alternative approach to deliver HBV into hepatocytes of immunocompetent mice bypassing conventional viral entry to establish a replicative reservoir [11–13]. Artificial HBV circles have been used in similar approaches [14, 15]. Only the ‘HBV circle’ model promotes HBV replication and the establishment of cccDNA-like molecules (cccDNA-LM) in immunocompetent mice [15].

We developed an HDI model using HBV genome monomers (1.0 mer) that can be easily adapted to any HBV genotype or serotype [16]. The model permits assessment of cccDNA-like structures in hepatocytes of immune-competent C57BL/6 mice. Additionally, to assess the efficacy of therapeutic drugs and their impact on cccDNA, we supplemented the in vivo model with in vitro studies of human primary liver organoids, derived from normal adult liver tissue [17, 18], which we infected with HBV [19]. These combined models were used to examine the ability of proapoptotic drugs to eliminate persistent HBV DNA reservoirs. We were particularly interested in testing drugs that preferentially promote extrinsic apoptosis of infected hepatocytes which we previously demonstrated [20, 21].

Death ligands such as tumor necrosis factor (TNF) can dichotomously promote cellular activation and survival or apoptosis. Inhibitor of apoptosis proteins (IAPs), which function as ubiquitin E3 ligases via their RING domain, constrain the ability of TNF to engage the apoptotic pathway and instead promote the activity of inflammatory pathways [22, 23]. When TNF binds to its cognate death receptor, TNFR1, IAP family members cellular IAP 1 (cIAP1) and cellular IAP 2 (cIAP2) are recruited to facilitate activation of NFκB and MAP kinases and promote pro-inflammatory signaling, cytokine production, and cell survival [24, 25]. IAP inhibitors were initially developed to promote the apoptosis of cancer cells [26]. These drugs, which can be bivalent or monovalent for their target, promote the auto-ubiquitination and degradation of cIAPs. This precludes activation of NFκB and allows for alternate activation of caspase-8-dependent apoptosis [27]. We have previously shown that HBV-infected hepatocytes have heightened sensitivity to TNF signaling, over and above uninfected hepatocytes, because HBV causes upregulation of TNFR1 protein expression in infected cells and promotes local TNF production (hepatocyte and/or immune cell driven) [11, 20]. A thorough dissection of bivalent and monovalent IAP inhibitors and their impact on episomal HBV has not been performed using robust in vitro and in vivo models and this is essential to guide clinical studies with a cure agenda. Importantly, monovalent compounds represent the most tractable route to the clinic given their oral bioavailability and apparent safety profile compared to bivalent IAP inhibitors.

In this study, we used novel preclinical HBV infection models, including our mouse model and primary human liver organoids, and found that monovalent IAP inhibitors have the capacity to eliminate HBV episomes. The combination of positive preclinical data and the excellent safety/tolerability profile of orally administered monovalent IAP inhibitors facilitate progression to clinical trials.

## Materials and methods

### Materials

Restriction enzymes were purchased from New England Biolabs (Ipswich, MA, USA); Reagents: ETV (Bristol-Myers Squibb, New York, NY, USA), Debio 1143 (Debiopharm, Lausanne. Switzerland), Birinapant (HY-16591, MedChemExpress, Monmouth Junction, NJ, USA), LCL-161 (HY-15518, MedChemExpress). Antibodies: HBsAg for WB (H166, Abbott, Chicago, IL, USA), HBcAg for WB (ID8, in house), cIAP1 for WB (ALX-803-335-C100, Enzo, New York, NY, USA), β-actin conjugated to HRP (5125S, Cell Signalling Technology, Danvers, MA, USA), HBcAg for liver IHC (B0586, Dako, Santa Clara, CA, USA), HBcAg for organoid IHC and IF (ab115992, Abcam, Cambridge, UK), cleaved caspase 3 for IF (C10423, Thermo Fisher Scientific, Waltham, MA, USA), DAPI for IF (D1306, Thermo Fisher Scientific).

### Cell lines and tissue culture

HepG2 cells (ATCC, Manassas, VA, USA) were passaged in MEM medium (Gibco, Waltham, MA, USA) containing 10% fetal calf serum (Bovogen, Melbourne, Australia) and supplemented with 100 U/ml Penicillin/Streptomycin (Gibco) and 2 mM l-glutamine (Gibco). HepG2-NTCP cells (kindly gifted by Prof. Stephan Urban, DZIF, Heidelberg, Germany) were passaged in DMEM medium (Gibco) containing 10% fetal calf serum (Bovogen) and supplemented with 100 U/ml Penicillin/Streptomycin (Gibco) and 5 µg/ml puromycin dihydrochloride (Gibco). HepAD38 cells (Gilead Sciences, Foster City, CA, USA) were passaged in DMEM/F-12 medium (Gibco) containing 10% fetal calf serum (Bovogen) and supplemented with 100 U/ml Penicillin/Streptomycin (Gibco) and 0.5 mg/ml Geneticin (Gibco).

### Generation and preparation of HBV plasmids

Linear 1.0 mers of HBV A2 and D3 were amplified from cell culture supernatant following transfection of hepatoma cells with HBV plasmid pAAV-HBV1.2 [12], or 1.3 mer genotype D3 [28], by using Gunther PCR [29] and primers P1 and P2 listed in Supplementary Table 1. The C2 1 mer was identical to the 1.3 mer C2 clone previously described [28]. The linear DNA was then inserted into pCR-XL-TOPO vector (Life Technologies, Carlsbad, CA, USA) for HBV A2 and HBV D3, or pUC18 (ThermoFisher Scientific) for HBV C2 according to the manufacturer’s protocol. Plasmids were sequenced using primers listed in Supplementary Table 1. Plasmids were amplified by transformation into One Shot Stbl3 chemically competent *E. coli* and grown at 37 °C overnight in Linder grain media, then growth was continued in lysogeny broth at 37 °C overnight. The EndoFree Plasmid Maxi Kit (Qiagen, Hilden, Germany) was used to extract the plasmid. The linear HBV-DNA 1.0 mers were isolated from the HBV A2 and HBV D3 plasmids by restriction endonuclease digestion with *SapI*, and from HBV C2 plasmid by digestion with *SapI* and *PvuII* (New England Biolabs). All reactions were cleaned up with the QIAquick PCR Purification kit (Qiagen).

### Mice and induction of HBV infection

All animal studies were performed in accordance with the requirements of the Australian Code for the Care and Use of Animals for Scientific Purposes. C57BL/6 (in house breeding) and gene-targeted TNF^−/−^ C57Bl/6 mice, previously characterized [30], were housed under specific pathogen-free conditions, with a 12 h light:dark cycle and room temperatures between 17 and 22 °C, in the Animal Research facilities. Mice of both genders, aged between 6 and 8 weeks were used for all experiments. Mice were grouped randomly. Sample size estimate for in vivo studies was based on past experience and confirmed using Prism 8 software version 8.4.3 (GraphPad, San Diego, CA, USA) based on anticipated effect sizes. 10 μg of HBV 1.0 mer total DNA was intravenously injected, within 5 s, via the tail vein in a volume of PBS equivalent to 8% v/w of the mouse body weight [31].

### Serum HBV DNA quantification

Submandibular bleeds (50–75 μl) were taken from mice once weekly after induction of infection for 8 weeks. The blood was allowed to clot and then the serum was separated from plasma by two rounds of centrifugation at 6000×*g* for 10 and 2 min, respectively, at room temperature. Viral DNA was extracted from 40 μl of serum using the Invisorb Virus DNA HTS 96 kit (Stratec, Birkenfield, Germany) according to manufacturer’s protocol and isolated DNA was eluted in 40 μl of elution buffer. HBV-DNA serum levels were quantified by quantitative real-time PCR using the FastStart Universal SYBR Green Master (Rox) Kit (Roche, Basel, Switzerland) on a LightCycler 480 II Machine (Roche) with absolute quantification software (Roche). A serial dilution of the linear HBV plasmid was used as internal standard, HBV D3 was linearized with *PstI* (New England Biolabs), HBV C2 with *HindIII* (New England Biolabs), and HBV-A2 was linearized with *SapI*. The limit of detection of serum HBV-DNA is 500 copies/ml.

### HBsAg and HBeAg quantification

Quantification of serum HBsAg and HBeAg were performed using electrochemiluminescence on the Cobas e411 analyser (Roche) as according to the manufacturer’s protocols through the Public Health Reference and Diagnostic Testing Laboratory Service at the Victorian Infectious Disease Reference Laboratory (VIDRL), Peter Doherty Institute for Infection and Immunity.

### Hematoxylin and Eosin staining, HBcAg, and cleaved Caspase-3 immunohistochemistry

Mouse livers were submerged in 10% buffered formalin and embedded in paraffin. 4–6 μM tissue sections were prepared and stained with hematoxylin and eosin (Leica, Wezlar, Gernany), anti-Caspase 3 cleaved (1:300, Cell Signalling Technology) and anti-HBcAg (1:500). Slides were scanned using a Panoramic SCAN II scanner (3D Histech, Budapest, Hungary). Images were analyzed in CaseViewer 2.8 (3D Histech).

### Murine HBV DNA isolation, Southern blotting, and cccDNA droplet digital PCR

Mouse livers were collected at 3 weeks post-induction of infection and snap frozen in liquid nitrogen. Snap-frozen liver tissue was homogenized in 1 ml buffer (50 mM Tris buffer, pH 7.4, 10 mM EDTA) using the Qiagen TissueLyserII (30 Hz, 5 min). Samples were mixed with 240 µl 10% SDS and 200 µl 2.5 M KCl and clarified by centrifugation at 4 °C for 10 min at full speed. The resulting supernatant was transferred to fresh tubes and subjected to 2× phenol and 1× chloroform extraction. DNA was left to precipitate at −20 °C overnight in 0.7× volume of isopropanol and 10 µg glycogen. DNA was pelleted by centrifugation for 10 min at full speed, washed with 70% ethanol, and finally resuspended in a volume of nuclease-free water. DNA samples were then split for multiple confirmatory treatments (e.g. EcoRI digest, ExoI/III digest). All samples were prepared in parallel and equivalent amounts analyzed by Southern blot as previously described [28]. HBV cccDNA levels were quantified using the Qx200 ddPCR system (Biorad, Hercules, CA, USA). The cccDNA assay consisted of two forward primers (DRF1: 5′-GTC TGT GCC TTC TCA TCT GC-3′ and CCC1: 5′-GCG GWC TCC CCG TCT GTG CC-3′), one reverse primer (CCC2: 5′-GTC CAT GCC CCA AAG CCA CC-3′), and amplified products were detected using a Taqman probe (5′-6FAM-CCG TGT GCA CTT CGC TTC ACC TCT GCA CG-BHQ1-3′) that spans the gap region. Each reaction contained 12.5 µl of 2× ddPCR™ Supermix for Probes (Biorad), 225 nM of DRF1, 225 nM of CCC1, 450 nM of CCC2, 250 nM of probe, and 2 µl of template. Exonuclease-treated templates were diluted 1:10 using nuclease-free water beforehand. Undigested templates were assayed alongside exonuclease-treated templates to determine levels of both cccDNA and other intermediate DNA species. Droplets were generated using an Automated Droplet Generator (Biorad) and samples were amplified using the following protocol on a C1000 Thermal Cycler (Biorad); 95 °C for 10 min, followed by 45 cycles of 94 °C for 30 s and 65 °C for 1 min, and a final cycle of 98 °C for 10 min (2.5 °C/s ramp was applied between each step). Amplified products were detected using a Qx200 Droplet Reader (Biorad) and analyzed using QuantaSoft™ Analysis Pro (Biorad). A normalization assay was performed to determine the number of genomic equivalents (GEq). A Mouse RPP30 Assay (Assay ID: dMmuCNS873761785) was purchased from Biorad and was performed according to manufacturer’s instructions. cccDNA levels were expressed as a ratio of cccDNA copies/GEq. The limit of detection is 1 cccDNA copy/2 mg of liver.

### HBV DNA isolation from cells and Southern blotting

Cells (60 mm dish) were lysed 5 days post-transfection in 600 μl of cccDNA Lysis buffer (10 mM Tris–HCl, pH 7.4, 10 mM EDTA, 0.5% SDS), detached using a cell lifter, and lysates were mixed with 200 μl of 2.5 M KCl. Samples were incubated at room temperature for 30 min then centrifuged for 20 min at room temperature to remove insoluble proteins. The resulting supernatant was subjected to 2× phenol and 1× chloroform extraction. DNA was precipitated at room temperature for 30 min with 2× volume room temperature ethanol and 6 μg glycogen. Following precipitation, the DNA was pelleted by centrifugation for 10 min, washed with 70% ethanol, and resuspended in nuclease-free water. Southern blot analysis performed as previously described [28].

### Northern blotting

Snap frozen mouse liver tissue was homogenized in 1 ml Trizol reagent using the Qiagen TissueLyserII before being stored at −70 °C. Before extraction, tubes were thawed on ice with frequent vortexing to ensure homogenization. Samples were then incubated at room temperature for 5 min before centrifugation at 4 °C for 10 min at 12,000×*g*. The resulting supernatant was mixed with 200 µl chloroform, incubated at RT for 2–3 min and centrifuged at 4 °C for 15 min at 12,000×*g*. The resulting top aqueous phase was transferred to fresh tubes and incubated with 500 µl isopropanol for 10 min at RT. Samples were centrifuged at 4 °C for 10 min at 12,000×*g*. The supernatant was aspirated and the pellet was washed with 80% ethanol (vortex, centrifuge 5 min at 4 °C, 7500×*g*). Ethanol was aspirated and pellet allowed to air-dry for 2–3 min, transferred to a 70 °C heat block and incubated for 2–3 min. The pellets were redissolved in 100 µl RNase-free water. RNA was further purified using RNeasy Kit (Qiagen) according to the manufacturer’s protocol and eluted in RNase-free water. Samples were heat-denatured in equal volume of NorthernMax-Gly sample loading dye (Invitrogen, Carlsbad, CA, USA) at 50 °C for 30 min prior to analysis by Northern blot as previously described [28].

### Western blot analysis cell culture HBc and HBs

Cells were lysed 5 days post-transfection in protein lysis buffer (150 mM NaCl, 50 mM Tris, (pH 7.5), 1% (v/v) NP-40) supplemented with Pierce Protease/Phosphatase Inhibitor Mini tablets (Thermo Fisher) at 4 °C for 15 min on a rocking platform. Cell lysates were collected into pre-chilled 1.5 ml tubes and centrifuged at 130,000×*g* for 20 min at 4 °C. The supernatant was transferred to fresh tubes and stored at −20 °C until required. Equivalent amounts of samples were boiled in Laemmli buffer for 5 min prior to SDS–PAGE. Lysates (50 μg protein per lane) were separated using 4–12% SDS/PAGE. Proteins were transferred onto nitrocellulose membranes and detected using primary and secondary antibodies. Antibodies used: 1D8 for HBcAg [32], H166 for HBsAg, kindly donated by Paul Coleman, Abbott Laboratories, Abbott Park, IL, USA.

### Western blot analysis mouse liver

Total liver protein lysates were prepared from 25 mg liver tissue that was homogenized in cell lysis buffer containing 20 mM Tris·HCl, pH 7.5, 135 mM NaCl, 1.5 mM Mg_2_Cl, 1 mM EGTA, 1% Triton X-100 (Sigma-Aldrich, St. Louis, MO, USA), 10% Glycerol (Ajax FineChem, Taren Point, Australia), EDTA-free protease inhibitor mixture tablets, and phosphatase inhibitor mixture tablets (Roche) using a tissue homogenizer (Tissue Lyser II; Qiagen). Lysates (50 μg protein per lane) were separated using 4–12% SDS/PAGE. Proteins were transferred onto nitrocellulose membranes and detected using primary and secondary antibodies. Antibodies used: rabbit anti-cIAP1 (1:1000; in house), mouse anti-XIAP (1:1000; MBLI, Woburn, MA, USA), goat anti-mouse (1:1500; Southern Biotech, Birmingham, AL, USA), and rabbit anti–β-actin (1:3000; Cell Signaling Technology, Danvers, MA, USA).

### IAP antagonists, ETV and LPS/GalN treatment in mice

ETV (Bristol-Myers Squibb, New York, NY, USA) was dissolved in peanut oil and given at 2 mg/kg daily via oral gavage for 14 days (day 7–21). Debio 1143 (provided by Debiopharm International SA, Lausanne, Switzerland) was dissolved in the manufacturer-supplied vehicle and given at 100 mg/kg daily via oral gavage for 14 days (day 7–21). Birinapant (MedChemExpress, Monmouth Junction, NJ, USA) was dissolved in DMSO and injected intraperitoneally weekly on day 7, 14, and 21 post-induction of infection at a concentration of 30 mg/kg. 100 ng of Lipopolysaccharide (Sigma-Aldrich) and 20 mg of d-galactosamine (Sigma-Aldrich) was dissolved in PBS and injected intraperitoneally.

### Alanine aminotransferase (ALT) and aspartate aminotransferase (AST) quantification

Serum levels of ALT and AST were measured using the activated ALT and AST assay on an architect c4000 analyzer (Abbott, Abbott Park, IL, USA).

### Generation of liver organoids and HBV infection

Liver organoids from healthy donors were isolated and cultured using the method previously described [17, 18] with minor modifications. In brief, liver specimens (1–2 cm^3^) were washed once with DMEM (Sigma-Aldrich) supplemented with 1% FCS and with 0.1% Penicillin Streptomycin (PS, Sigma-Aldrich), minced and incubated at 37 °C with the digestion solution (collagenase 2.5 mg/ml in EBSS). Incubation was performed for 30 min, further mincing and mixing the tissue every 10 min. To recover the cells, digestion solution was passed through a 70 µM strainer in a 50 ml tube (GreinerBio, Kremsmünster, Austria) and washed with 45 ml Advanced DMEM (Gibco) supplemented with 1% PS, 10 mM HEPES (Gibco), and 1% GlutaMax (Gibco), henceforth Ad+ ++. Partially digested tissue was recovered from the strainer and further incubated with TrypLE Express (Thermo Fisher Scientific) for 15 min at 37 °C. Cells obtained from the first and second digestion were pooled together and washed twice with Ad+++. After the second centrifugation (200×*g*, 5 min) cells were counted, mixed with an appropriate amount of BME solution (2/3 Basement Membrane Extract, Type 2, Pathclear (Trevigen, Inc., Gaithersburg, MD, USA) diluted with 1/3 Ad+++) and seeded in 25 µl drops containing 10,000–15,000 cells in 48-well suspension plates (GreinerBio). After BME solution had solidified, wells were filled with 250 µl of human liver organoid isolation medium consisting Ad+++ supplemented of 1× B27 supplement without retinoic acid (Gibco), 1× N2 supplement (Gibco), 1.25 mM N-acetyl-l-cysteine (Sigma-Aldrich), 20% (vol/vol) Rspo-1-conditioned medium [33], 1.25% (vol/vol) Wnt3a-conditioned medium (Barker et al., 2010), 10 mM nicotinamide (Sigma-Aldrich), 10 nM recombinant human (Leu15)-gastrin I (Sigma-Aldrich), 50 ng/ml recombinant human EGF (Peprotech, Rocky Hill, NJ, USA), 100 ng/ml recombinant human FGF10 (Peprotech), 25 ng/ml recombinant human HGF (Peprotech), 10 μM Forskolin (Sigma-Aldrich), 5 μM A8301 (Tocris, Bristol, UK), 25 ng/ml Noggin (Peprotech) and 10 μM Y27632 (Sigma-Aldrich). After 1 week, isolation media was changed to human liver expansion media (EM; Ad+++ supplemented of 1× B27 supplement without retinoic acid (Gibco), 1× N2 supplement (Gibco), 1.25 mM N-acetyl-l-cysteine (Sigma), 20% (vol/vol) Rspo-1-conditioned medium, 1.25% (vol/vol) Wnt3a-conditioned medium [34], 10 mM nicotinamide (Sigma-Aldrich), 10 nM recombinant human (Leu15)-gastrin I (Sigma-Aldrich), 50 ng/ml recombinant human EGF (Peprotech), 100 ng/ml recombinant human FGF10 (Peprotech), 25 ng/ml recombinant human HGF (Peprotech), 10 μM Forskolin (Sigma-Aldrich) and 5 μM A8301 (Tocris) [17].

EM was changed twice a week, and cultures were split every 7–10 days according to organoid density. For passaging (1:4–1:8, depending on the growth rate of the culture), organoids were resuspended in 10 ml Ad+++, incubated in ice for 10 min and collected by centrifugation (5 min at 200×*g*). Subsequently, organoids were incubated for 1–2 min in TrypLE Express at room temperature and mechanically disrupted by pipetting. After a further wash in Ad+++, cells were resuspended in BME solution and seeded in 24 or 48 wells suspension plates. After BME solution had solidified, wells were filled with 500 µl (24 wells) or 250 µl (48 wells) of human liver organoid expansion medium. Before differentiation organoids were kept for 4 days in EM without Wnt3a-conditioned medium supplemented with 25 ng/ml of BMP7 (Peprotech). Hepatic differentiation was induced by culturing human liver organoids in differentiation medium (DM; Ad+++ supplemented with 1X B27 supplement without retinoic acid, 1× N2 supplement, 1 mM N-acetylcysteine, 10 nM recombinant human [Leu15]-gastrin I, 50 ng/ml recombinant human EGF, 25 ng/ml recombinant human HGF, 0.5 μM A83-01, 10 μM DAPT (Sigma-Aldrich), 3 μM dexamethasone (Sigma-Aldrich), 25 ng/ml BMP7 and 100 ng/ml recombinant human FGF19 (Peprotech)). HepG2.2.15 cells, a HepG2 derived cells line stably transfected with full-length HBV (kindly provided by Prof. Bart Haagmans, Erasmus MC) were cultured in DMEM medium (Gibco) supplemented with 10% fetal bovine serum (Gibco) and 1% penicillin/streptomycin. For virus production, 3 × 10^6^ cells were plated in collagen coated 10 cm plates, cultured in supplemented DMEM until confluency and subsequently in Ad+++ for 4 days. The supernatant of HepG2.2.15 cells was then collected, filtered, and concentrated using the PEG Virus Precipitation Kit (Abcam, Cambridge, UK) following the manufacturer’s instructions. Precipitated virus was aliquoted and stored at −80 °C until use. As the negative control, an aliquot of the virus equivalent to the inoculum was inactivated by incubation at 100 °C for 30 min. Human liver organoids were resuspended using either active virus or heat-inactivated control at an MOI of 1–10*10^3^ copies HBV DNA/organoid, transferred to 24 wells plate, and centrifuged for 1 h at 600×*g*. Following spin-inoculation, plates were incubated at 37 °C for 5 h and then seeded in BME following the culturing protocol. After BME solution has solidified, liver organoids were maintained in EM for 16 h, washed 4 times with Ad+++ and cultured EM or DM as indicated.

### HBV DNA quantification organoids

DNA was extracted from culture supernatants using the QIAamp MinElute Virus Spin Kit following manufacturer’s instructions and was analyzed in duplicate using a TaqMan-based qPCR assay. For each reaction, a 12 µl mixture was prepared containing 5 µl LightCycler^®^480 Probes Master (Roche), 100 µM forward primer (5′-GCAACTTTTTCACCTCTGCCT A-3′) and reverse primer (5′-AGTAACTCCACAGTAGCTCCAAATT-3′), 0.075 µl of 50 µM probe (FAM-TTCAAGCCTCCAAGCTGTGCCTTGGGTGGC-TAMRA), and 4 µl DNA. Each PCR reaction included a standard curve made of dilutions of a plasmid containing the full-length HBV genome ranging from 2 to 2 × 10^5^ copies of plasmid.

### Immunofluorescence assay organoids

Human liver organoids were collected and washed three times with cold Ad+++ to remove BME and treated with 2 µM CellEvent™ Caspase-3/7 Green Detection Reagent for 30 min at room temperature. Organoids were then fixed with 4% paraformaldehyde for 30 min in ice followed by treatment with 0.1 M glycine in PBS + 2% FCS for 15 min on ice. They were then permeabilized using 0.3% Triton X-100 (Sigma-Aldrich) in PBS + 2% FCS for 30 min at room temperature. Specimens were incubated for 2 h at room temperature in PBS plus 0.5% FCS, 0.3% triton, 1% BSA 1% DMSO. Following blocking, human liver organoids were incubated overnight with primary antibodies (anti-HBcAg 1:200, Abcam) diluted in PBS + 10% blocking buffer. After extensive washing, human liver organoids were stained with appropriate Alexa Fluor dye-conjugated secondary antibodies (Life Technologies). Nuclei were stained with DAPI (1:1000, Thermo Fisher Scientific). Immunofluorescence images were acquired using an SP5 confocal microscope (Leica, Wetzlar, Germany). All phase-contrast pictures were acquired using a Leica DMIL microscope and a DFC420C camera. Images were analyzed and processed using Fiji/ImageJ (NIH, Bethesda, MD, USA).

### Statistical analysis

The details of experiments, including statistical tests used, the number of experiments, as well as dispersion and precision measures, are stated in individual figure legends. Mice were grouped randomly. Prism 8 software version 8.4.3 (GraphPad, San Diego, CA, USA) was used to perform statistical tests and to determine the sample size for experiments based on anticipated effect sizes. Independent groups were compared by unpaired two-tailed *t* tests for parametric data and Holm–Sidak correction was applied for multiple *t* tests. *P value < 0.05 was defined as statistically significant. Data used are mean ± standard error of the mean (s.e.m.).

## Results

### Single length HBV DNA ‘monomers’ generate cccDNA-like molecules in HepG2 cells and permit viral replication

**Fig. 1:**
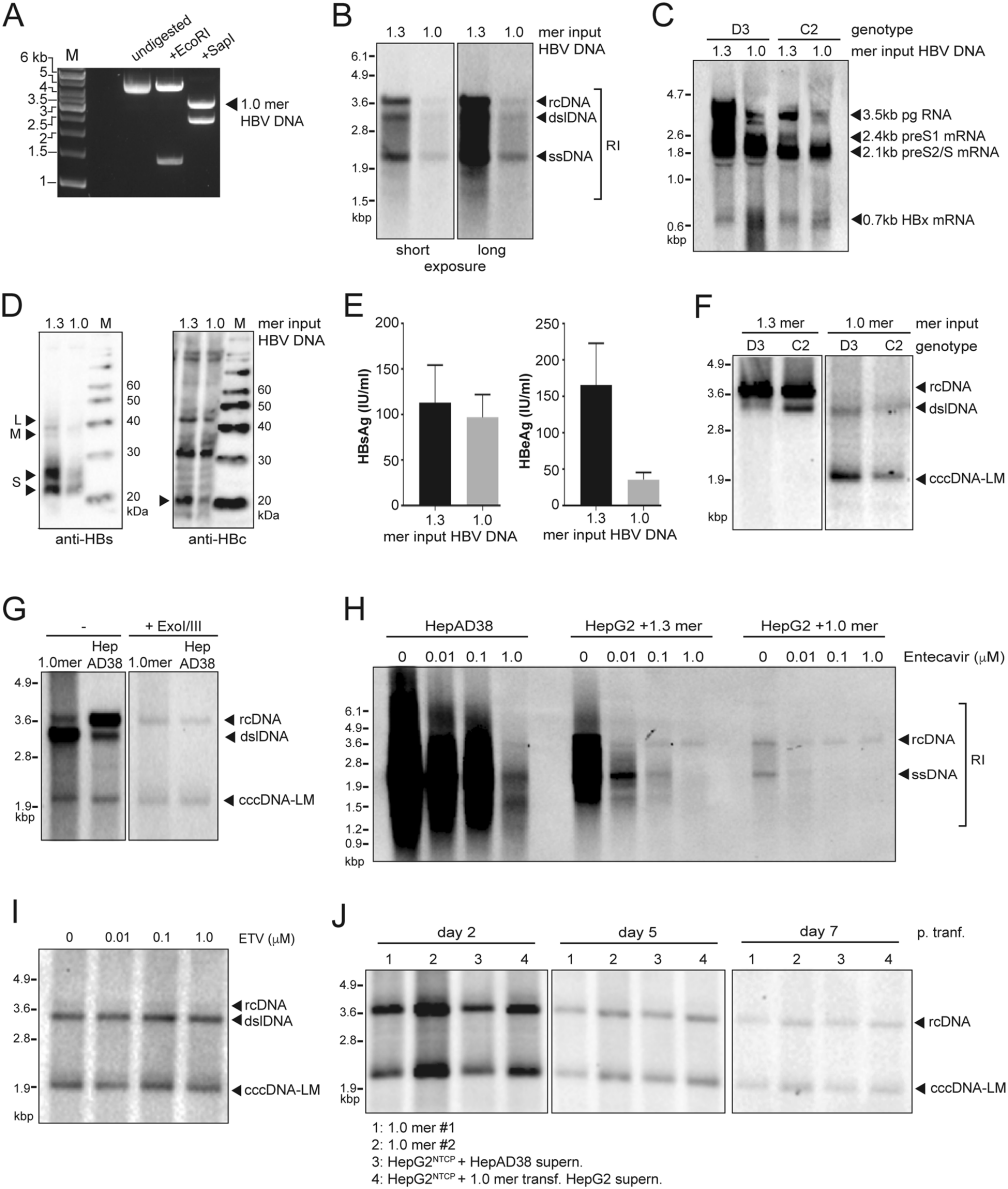
HBV 1.0 mers promote HBV replication and facilitates formation of cccDNA-like molecules in HepG2 cells. **A** Agarose gel electrophoresis of HBV 1.0mer DNA (genotype D3) digested with indicated restriction endonucleases, 1.0 g per lane, M, DNA ladder marker. **B** Southern blot analysis of intracellular HBV core-associated HBV DNA extracted from HepG2 cells at day 5 post-transfection of wildtype HBV 1.3 plasmid or linearized 1.0 mer DNA (genotype D3). **C** Northern blot analysis of intracellular HBV RNA from HepG2 cells 5 days post-transfection with wildtype HBV 1.3 plasmid or linearized 1.0 mer DNA. **D** Western blot analysis of intracellular HBsAg (left) and HBcAg (right), indicated by black arrows from total HepG2 lysates at day 5 post-transfection with HBV 1.3 plasmid or linearized 1.0 mer DNA (genotype D3). **E** Quantitative analysis of HBsAg (left) and HBeAg (right) from the supernatant of HepG2 cells at day 5 post-transfection with HBV 1.3 plasmid or linearized 1.0 mer DNA (genotype D3). Data are represented as mean ± s.e.m. **F** Southern blot analysis of HBV DNA extracted by Hirt-lysis from HepG2 cells at day 5 post transfection with HBV 1.3 plasmid or linearized 1.0 mer DNA. **G** Southern blot analysis of HBV DNA extracted by Hirt-lysis from HepG2 cells at day 5 post transfection of wildtype 1.0mer DNA (genotype D3) or from replication induced HepAD38 cells 7 days post seeding, undigested or Exo I/III digested. **H** Southern blot analysis of intracellular core-associated HBV DNA from HepAD38 cells at day 7 post seeding or from HepG2 cells at day 5 post transfection with HBV1.3mer plasmid or linearized 1.0 mer. Cells were maintained in the presence of indicated concentrations of Entecavir. **I** Southern blot analysis of HBV DNA extracted by Hirt-lysis from HepG2 cells at day 5 post transfection with linearized 1.0 mer HBV DNA, after treatment with entecavir at indicated concentrations. **J** Southern blot analysis of HBV DNA extracted by Hirt-lysis at indicated timepoints from HepG2 cells transfected with HBV 1.0 mer genotype D3 (lane 1 + 2), transgenic HepG2^NTCP^ cells inoculated with HBV containing supernatant of HepAD38 cells (lane 3) or transgenic HepG2^NTCP^ cells inoculated with HBV containing supernatant of HBV 1.0 mer (genotype D3) transfected HepG2 cells. Results are representative of three independent experiments.

We first assessed the ability of linearized HBV 1.0 mers to form cccDNA in HepG2 cells. We PCR-amplified HBV DNA template molecules based on previously characterized clones isolated from patient serum (genotype C, kindly gifted by Yu-Mei Wen, Shanghai Medical Centre, Fudan University) or cell culture-derived (genotypes A2 and D3) [28] and performed *SapI* endonuclease digestion according to ‘Gunther-PCR’ protocols [29] (Fig. 1A). HepG2 cells were transfected with genotype D3 and C2 HBV monomers and compared to cells transfected with a plasmid containing a 1.3 overlength HBV DNA construct [35]. HBV replicative intermediates (RI), including relaxed-circular (rc), double-stranded linear (dsl), and single-stranded (ss) DNA were identified in HBV monomer-transfected cells (Fig. 1B) along with HBV pre- and subgenomic RNAs (Fig. 1C). HBV surface antigen (HBsAg) and HBV *core* protein (HBcAg) were detected in HBV monomer transfected cells (Fig. 1D) and HBsAg and HBeAg were also detected in the supernatant of these cultures (Fig. 1E).

Importantly, following modified Hirt lysis protein-free viral DNA extraction [35], we were able to identify cccDNA-like molecules by Southern blot analysis in HepG2 cells transfected with genotype D3 and C2 1.0 mers but not in cells transfected with plasmid 1.3 mers (Fig. 1F). Exonuclease (Exo) I/III digestion of extracted DNA is able to distinguish between ‘leftover’ ssDNA, dsDNA (including rcDNA that has a free 3’ end on both DNA strands) and cccDNA [36]^.^ The cccDNA like structures extracted from HepG2 cells transfected with 1mers were resistant to Exo I/III digestion (Fig. 1G). HepAD38 cells, an inducible HBV hepatoma cell line expressing cccDNA [37] were used as positive control (Fig. 1G). The nucleoside analog ETV is known to block production of rcDNA but has negligible impact on cccDNA levels. We found that doses of ETV able to block rcDNA (Fig. 1H), had no effect on the levels of cccDNA-like molecules in HBV 1.0 mer transfected HepG2 cells (Fig. 1I). cccDNA like molecules were detectable for at least 7 days in HepG2 cells transfected with HBV 1.0 mers (Fig. 1J). Collectively the data showed that HBV 1.0 mers, of various genotypes generated cccDNA like molecules in HepG2 and underscored the rationale for pursuing this method to establish cccDNA in vivo in mice.

### HDI of HBV 1.0 mers generate a hepatic reservoir of cccDNA-like molecules in C57BL/6 mice

**Fig. 2:**
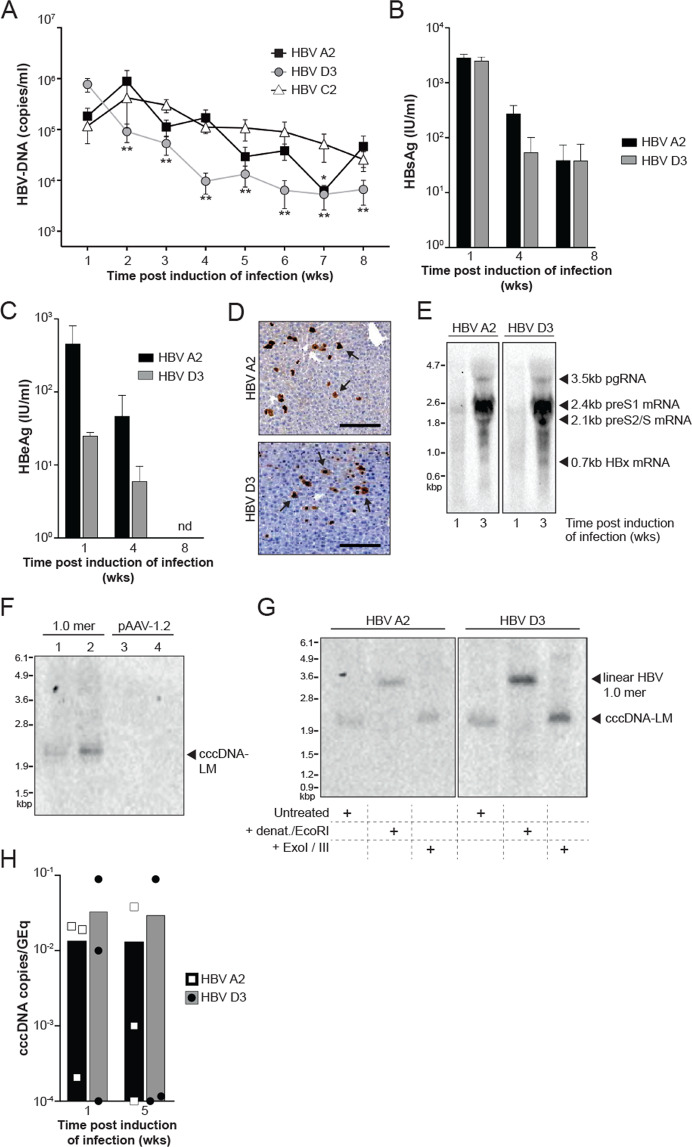
HBV replication and formation of cccDNA-Like molecules in C57BL/6 mice. **A** Serial measurement of serum HBV DNA levels in C57BL/6 mice after induction of infection with HBV 1.0 mers. Data are represented as mean ± s.e.m. from three independent experiments (*n* = 15 for genotype A2, *n* = 12 for genotype D3, *n* = 15 for genotype C2). Statistical analyses using unpaired two-tailed *t* tests were performed comparing each week with week 1. *P < 0.05, **P < 0.01. **B** Quantitative analysis of HBsAg in serum of C57BL/6 mice at indicated time points after induction of infection with HBV 1.0 mers. Data are represented as mean ± s.e.m. from two independent experiments (*n* = 12 for genotype A2, *n* = 11 for genotype D3). **C** Quantitative analysis of HBeAg in serum of C57BL/6 mice at indicated timepoints after induction of infection with HBV 1.0 mers of indicated genotypes. Data are represented as mean ± s.e.m. from two independent experiments (*n* = 12 for genotype A2, *n* = 11 for genotype D3). nd = non-detectable. **D** Immunohistochemistry staining of HBV core antigen positive cells in liver sections of C57BL/6 mice one week after induction of infection with the indicated HBV genotypes, arrows indicate HBcAg-positive stained liver cells, *n* = 6 per group, scale bars: 125 μm. Data is representative of two independent experiments. **E** Northern blot analysis of total liver HBV RNA from C57BL/6 mice at indicated time points after induction of HBV infection with indicated genotypes. Data is representative of two independent experiments. **F** Southern blot analysis of HBV DNA extracted by Hirt-lysis from total liver of C57BL/6 mice 3 weeks post induction of infection with HBV 1.0 mer, genotype D3 (lanes 1 and 2) in comparison to mice transfected with pAAV-HBV1.2 plasmid DNA, genotype A2 (lanes 3 and 4). Data is representative of two independent experiments. **G** Southern blot analysis of HBV DNA extracted by Hirt-lysis and exposed to heat denaturation and EcoRI digest or Exo I/III digest in comparison to untreated DNA from total liver of C57BL/6 mice 3 weeks post induction of infection with HBV 1.0 mer (genotype A2 or D3). Results are representative of three independent experiments. **H** Quantification of HBV cccDNA from total liver of C57BL/6 mice 1- and 5-weeks post induction of infection with HBV 1.0 mer (genotype A2 and D3) and treatment with specified compounds. Data are represented as mean (*n* = 3 per time point). HBV cccDNA levels expressed as a ratio of cccDNA copies per genomic equivalents (GEq). The limit of detection is 1 cccDNA copy/2 mg of liver.

To establish HBV persistence in immunocompetent C57BL/6 mice, we hydrodynamically injected PCR amplified and linearized HBV 1.0 mers of different genotypes via the tail vein as previously described [11]. We quantified HBV DNA levels over 8 weeks in the serum of mice after induction of infection with HBV genotype A2, D3, and C2 (Fig. 2A). With a focus on the analysis of genotypes A2 and D3, we found a gradual decline in HBsAg and HBeAg levels in the serum of HBV mice mirroring the decline in DNA levels (Fig. 2B, C). HBcAg positive hepatocytes were detected using immunohistochemistry (Fig. 2D) and the presence of HBV pre- and subgenomic RNAs in the liver of mice was confirmed at week 1 and 3 post induction of infection by Northern blot analysis (Fig. 2E). Importantly, in animals injected with HBV 1.0 mers we identified HBV DNA molecules that corresponded to the molecular size of cccDNA in contrast to mice hydrodynamically injected with pAAV-HBV1.2 DNA [11] (Fig. 2F). We confirmed cccDNA-like characteristics as defined by a gel shift to a 3.2 kb dslDNA position after heat denaturation in combination with restriction endonuclease EcoRI digestion and resistance of cccDNA-LM to Exo I/III treatment (Fig. 2G). We also used digital drop PCR to examine the kinetics of liver cccDNA over time in mice infected with HBV A2 and D3 (Fig. 2H). The very small input volumes required for this technique compromised the limit of detection but overall, the trend shows the persistence of cccDNA. These data demonstrated that PCR amplified HBV 1.0 mers, hydrodynamically injected into C57BL/6 mice, can generate cccDNA-like molecules in the liver and can sustain prolonged viral replication. This provides a valuable preclinical platform that can be used to examine the efficacy of drugs in eliminating cccDNA.

### The monovalent IAP antagonist Debio 1143 promotes elimination of HBV infection in vivo

A tenable strategy to remove cccDNA could involve progressive and preferential induction of apoptosis in infected hepatocytes to cause the destruction of DNA. We have previously shown that IAP inhibitors can promote clearance of HBV in a preclinical model, but their impact on cccDNA has not been examined [20]. Using our novel in vivo preclinical model we investigated the therapeutic efficacy of the clinical-stage orally administered monovalent IAP inhibitor Debio 1143 (xevinapant) and its impact on cccDNA [38]. In clinical cancer trials, Debio 1143 was shown to be highly efficacious in decreasing cIAP1 in patient-derived PBMCs at doses above 80 mg and it was well tolerated up to 900 mg [39, 40]. For treatment of C57BL/6 mice, we used oral single daily dose of 100 mg/kg that approximates the exposure measured in human subjects treated with a dose of 200 mg, which is the recommended dose currently explored in cancer patients [41].

**Fig. 3:**
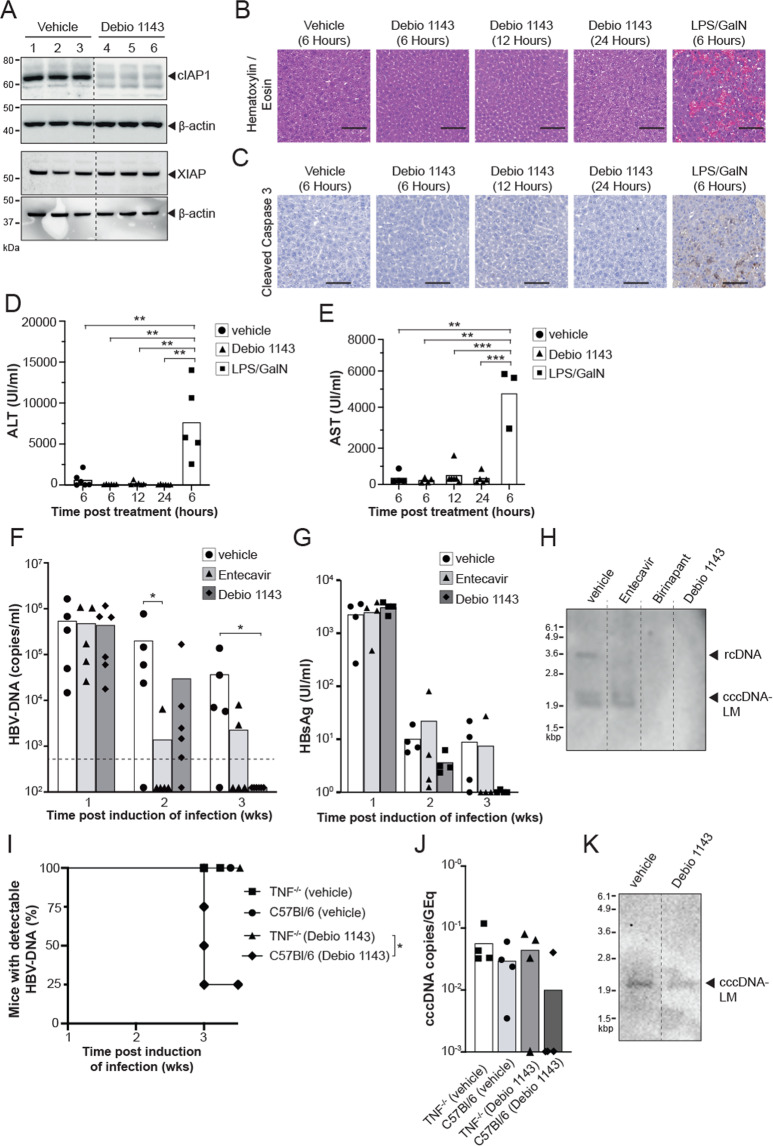
Debio 1143 promotes clearance of HBV infection in C57BL/6 mice. **A** Western blot analysis of cIAP1, XIAP (black arrow), and β-Actin protein levels in the liver of naive C57BL/6 mice 18 h after a single dose of vehicle or Debio 1143 treatment (*n* = 3 mice per group, indicated by numbers above blots). Data is representative of three independent experiments. **B** Hematoxylin and eosin staining of liver sections of uninfected C57Bl/6 mice at indicated timepoints after a single dose of vehicle, Debio 1143 or LPS/GalN (*n* = 3 mice per group). Data is representative of two independent experiments. Scale bars = 100 µM. **C** Immunohistochemistry staining of cleaved caspase-3 in liver sections of uninfected C57Bl/6 mice at indicated timepoints after a single dose of vehicle, Debio 1143 or LPS/GalN. (*n* = 3 mice per group). Data is representative of two independent experiments. Scale bars = 100 µM. **D** Serum alanine aminotransferase (ALT) levels at indicated timepoints after a single dose of vehicle, Debio 1143 or LPS/GalN. Data are represented as mean (*n* = 5–6). Statistical analyses using unpaired two-tailed *t* tests were performed **P < 0.01. **E** Serum aspartate aminotransferase (AST) levels at indicated timepoints after a single dose of vehicle, Debio 1143 or LPS/GalN. Data are represented as mean (*n* = 3–6). Statistical analyses using unpaired two-tailed *t* tests were performed **P < 0.01, ***P < 0.001. **F** Serial measurement of serum HBV DNA levels in C57BL/6 mice after induction of HBV infection with HBV 1.0 mers (genotype D3) and treatment with specified compounds at indicated timepoints. Dashed line indicates assay detection limit of 500 HBV DNA copies/ml. Representative data of two independent experiments shown as mean (*n* = 5 for vehicle, *n* = 5 for Entecavir, *n* = 6 for Debio 1143). Statistical analyses using unpaired two-tailed *t* tests for parametric data and Holm–Sidak correction for multiple *t* tests was performed. *P < 0.05. **G** Measurement of serum HbsAg in C57BL/6 mice after induction of HBV infection with HBV 1.0 mers (genotype D3) and treatment with specified compounds at indicated timepoints. **H** Southern blot analysis of HBV DNA extracted by Hirt-lysis from total liver of individual C57BL/6 mice 3 weeks post induction of infection with HBV 1.0 mer (genotype D3) and treatment with specified compounds. Results are representative from three independent experiments. **I** Proportion of animals and time when TNF^−/−^ or C57BL/6 mice after induction of HBV infection with HBV 1.0 mers (genotype D3) and treatment with Debio 1143 or vehicle first achieved an undetectable serum HBV DNA level. Data are represented as mean (*n* = 4). Statistical analyses using log-rank Mantel–Cox test was performed *P < 0.05. **J** Quantification of HBV cccDNA from total liver of individual TNF^−/−^ and C57BL/6 mice 3 weeks post induction of infection with HBV 1.0 mer (genotype D3) and treatment with specified compounds. Data are represented as mean (*n* = 4 per group). HBV cccDNA levels expressed as a ratio of cccDNA copies per genomic equivalents (GEq). The limit of detection is 1 cccDNA copy/ 2 mg of liver. **K** Southern blot analysis of HBV DNA extracted by Hirt-lysis from total liver of individual TNF^−/−^ mice 3 weeks post induction of infection with HBV 1.0 mer (genotype D3) and treatment with specified compounds.

IAP inhibitors promote the degradation of cIAPs through induction of autoubiquitination and proteasomal degradation. We examined the specific degradation of cIAP1 and XIAP proteins in naïve animals treated with a single oral dose of Debio 1143. We confirmed efficient degradation of cIAP1, 18 h after dosing, but not XIAP by Western blot analysis (Fig. 3A). To confirm Debio 1143 was not causing non-specific destruction of hepatocytes, livers were examined at 6, 12, and 24 hours post-treatment with Debio 1143 and compared to livers from mice treated with lipopolysaccharide (LPS) and l-galactosamine (GalN). LPS and GalN cause systemic release of TNF to induce non-specific apoptosis of hepatocytes [42]. Hematoxylin and Eosin staining and staining for cleaved caspase 3, a classical marker of apoptosis, confirms the extensive hepatocyte apoptosis induced by LPS and GalN which is not seen comparable to the preferentially targeted killing of hepatocytes seen in HBV-infected mice treated with Debio 1143 (Fig. 3B, C). This is further confirmed by examining serum levels of ALT and AST, which are both markers of liver damage. Mice injected with LPS and GalN had significantly higher serum AST and ALT levels as compared with mice treated with Debio 1143 (Fig. 3D, E). We next induced HBV infection in C57BL/6 mice with HBV 1.0 mers (genotype D3) and one week after infection we commenced a 2-week treatment course with daily oral Debio 1143. Mice receiving a vehicle control and daily ETV (2 mg/kg), the clinical standard of care nucleoside analog presently used to suppress HBV replication, were used as controls. Baseline HBV DNA levels in the serum of mice were determined immediately before treatment and then weekly by quantitative real time-PCR (qRT-PCR). Debio 1143 and ETV treatment caused a rapid reduction in serum HBV DNA levels after 1 week of treatment. All mice treated with Debio 1143 had undetectable serum HBV DNA levels after 2 weeks of treatment (Fig. 3F), and the majority had undetectable HBsAg serum levels (Fig. 3G), consistent with the induction of apoptosis in HBV-infected cells as demonstrated previously [20]. To determine if Debio 1143 can eliminate cccDNA-LM in hepatocytes, we performed Southern blot analysis on livers harvested from mice after 2 weeks of treatment (Fig. 3H). We found that doses of ETV, Debio 1143, or the bivalent IAP inhibitor birinapant were all able to eliminate rcDNA compared to vehicle-treated animals, but strikingly, we were not able to detect any cccDNA-LM in the liver of mice treated with IAP inhibitors, Debio 1143 or birinapant, compared to vehicle or ETV-treated animals (Fig. 3H). These findings revealed that both, monovalent and bivalent clinical stage IAP inhibitors (Debio 1143 and birinapant, respectively), can completely eliminate the HBV reservoir of infection, including cccDNA, in an in vivo model. To confirm that IAP inhibitor, Debio 1143, was utilizing the TNF pathway to cause apoptosis in HBV-infected cells, TNF^−/−^ and C57Bl/6 mice were infected with HBV 1.0 mer (genotype D3) and one week after infection we commenced a 2-week treatment course with daily oral Debio 1143 or vehicle (Fig. 3I). The majority of C57Bl/6 mice, treated with Debio 1143 had cleared the virus, whereas TNF^−/−^ mice treated with Debio 1143 and both vehicle-treated groups, remained infected. The cccDNA-LM levels were quantified by ddPCR (Fig. 3J) and confirmed by southern blot analysis (Fig. 3K). We found that Debio 1143 was not able to clear cccDNA-LM in the majority of TNF^−/−^ mice confirming Debio 1143 is utilizing the TNF pathway to cause apoptosis in HBV-infected cells and eliminate the cccDNA-LM.

### The monovalent IAP antagonist LCL-161 promotes elimination of HBV infection in primary human liver organoids

**Fig. 4:**
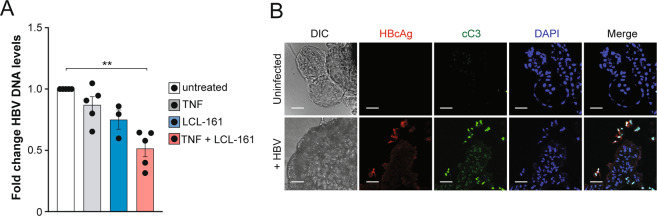
LCL-161 selectively induces TNF mediated apoptosis of HBV infected primary human liver organoids. **A** HBV DNA levels in supernatant of HBV-infected liver organoid cultures 4 days after indicated treatment with 10 μM LCL-161, 200 ng/ml recombinant TNF or the combinaton of both (8 days after infection), identical donor derived organoid cultures were used in all groups (*n* = 3 for LCL-161 only group, *n* = 5 for all other groups). Data are represented as mean ± s.e.m. and normalized to untreated organoid culture. Statistical analyses using unpaired two-tailed *t* tests for parametric data was performed. **P < 0.01. **B** Immunofluorescence staining of differentiated liver organoids, uninfected or infected with HBV and treated with 10 μM LCL-161 and 200 ng/ml TNF for 6 h on day 6 post infection. Scale bars: 50 μm. Donor-derived organoid cultures with *n* = 2 for uninfected and *n* = 4 for uninfected groups were used. DIC differential interference contrast, cC3 cleaved caspase 3. Results are representative from three independent experiments.

To gain a deeper and broader understanding regarding the efficacy of monovalent IAP inhibitors in eliminating HBV DNA, we tested another monovalent compound LCL-161 [43, 44] in an in vitro human liver organoid model to complement the data obtained with Debio 1143 in our animal model. We infected primary liver organoid cultures with HBV isolated from the supernatant of stably HBV-expressing transgenic hepatoma cells HepG2 2.15. Four days after infection, liver organoid cultures from five different donors were treated with LCL-161, recombinant TNF or the combination of both, and HBV DNA levels in culture supernatants were quantified after 4 days of treatment using qRT-PCR. Relative to HBV DNA levels from untreated liver organoids, there was a 50% reduction of HBV DNA in cultured liver organoids treated with LCL-161 and exogenous TNF. TNF and LCL-161 alone had limited effects on HBV DNA levels (Fig. 4A). These findings suggested that LCL-161-mediated degradation of IAPs in the presence of exogenous TNF-induced killing of HBV-infected liver organoid cells. To determine if LCL-161 was preferentially killing infected cells, we analyzed the levels of cleaved caspase 3 (cC3), in HBV infected and uninfected organoids by immunofluorescence imaging. Combined LCL-161 and TNF treatment caused caspase 3 cleavage predominantly in cells expressing core protein (HBcAg) (Fig. 4B). The findings suggest that the combination of LCL-161 and TNF selectively leads to the death of HBV-infected liver organoid cells causing a reduction in HBV-DNA levels, without inducing cell death in uninfected cells.

## Discussion

A cure for HBV infection is a priority to reduce disease related morbidity and mortality to obviate the need for lifelong antiviral therapy in people with chronic active HBV hepatitis [3]. The major obstacle for curative therapies is an inability to effectively remove persistent HBV cccDNA in the liver. We have previously shown that IAP inhibitors can be used to selectively induce the death of HBV-infected hepatocytes [20]. Here, we showed that the well-tolerated orally administered monovalent IAP inhibitors, Debio 1143 and LCL-161 [39, 40, 43, 44], are capable of eliminating cccDNA in an animal model and can selectively kill human HBV-infected primary liver organoid cells and inhibit HBV replication, respectively.

Our animal model that utilized linearized single length HBV can be easily adapted to any genotype or variant. The use of C57BL/6 mice permits analysis of many gene-targeted animals that have been generated on this background. Furthermore, this immunocompetent model will allow examination of immune-based anti-HBV therapies and their impact on cccDNA.

To underscore the veracity of our results we used two monovalent IAP inhibitors across two models. Collectively using Debio 1143 and LCL-161, and our two HBV models, we demonstrated the ability of monovalent IAP inhibitors to eradicate cccDNA in vivo and the ability of these compounds to preferentially induce apoptosis of HBV infected human hepatocytes. Our data aligns with previous work elucidating the mechanisms through which IAP inhibitors eliminate infected cells and their intracellular pathogens [20]. The fundamental advance here is the demonstration that this strategy can be employed to remove persistent HBV cccDNA reservoirs in the liver.

This important discovery together with the very favorable safety profile of monovalent IAP inhibitors underscores the opportunity to progress this class of drugs to clinical trials and examine therapeutic efficacy in the treatment of chronic HBV infection. We speculate, as was the case for bivalent IAP inhibitors, that these compounds can be combined with existing standard-of-care antiviral treatments. A crucial development here is that the apparent safety profile of orally bioavailable monovalent IAP inhibitors does not compromise their efficacy in clearing HBV infection compared to bivalent compounds.

## Supplementary information

Supplementary Table 1

Supplementary Table 1 figure legend
