# Supplementary material for: Clinical stage drugs targeting inhibitor of apoptosis proteins purge episomal Hepatitis B viral genome in preclinical models
Source: Cell Death Dis. 2021 Jun 23;12(7):641. doi: 10.1038/s41419-021-03924-0 (PMC8222287; doi:10.1038/s41419-021-03924-0)
Supplement: Supplementary file 2 — Supplementary Table 1 figure legend [file 41419_2021_3924_MOESM2_ESM.docx]

**Supplementary data:**

**Supplementary Table 1: Primer regions used for qPCR and sequencing of HBV-DNA**

Nucleotides are given in relation to the Hepatitis B virus genome (GenBank ID: NC_003977.2).
